# Atom-level descriptors and explainable prediction of iron carbide nanoparticles' cytotoxicity *via* the Enalos Cloud platform

**DOI:** 10.1039/d5na00549c

**Published:** 2025-11-25

**Authors:** Maria Antoniou, Dimitra-Danai Varsou, Andreas Tsoumanis, Georgia Melagraki, Iseult Lynch, Antreas Afantitis

**Affiliations:** a Department of Nanoinformatics, NovaMechanics Ltd. Nicosia 1046 Cyprus afantitis@novamechanics.com; b Computation-Based Science and Technology Research Center, The Cyprus Institute Nicosia 2121 Cyprus; c Entelos Institute Larnaca 6059 Cyprus; d Department of NanoInformatics NovaMechanics MIKE Piraeus 18545 Greece; e Division of Physical Sciences & Applications, Hellenic Military Academy 16672 Vari Greece; f School of Geography, Earth and Environmental Sciences, University of Birmingham Edgbaston Birmingham B15 2TT UK; g Department of Pharmacy, Frederick University Nicosia 1036 Cyprus

## Abstract

Iron carbide nanoparticles (ICNPs), a distinct type of magnetic nanostructure, have been proposed as novel candidate therapeutic agents for a wide range of biomedical applications, yet their biocompatibility remains a critical concern for their safe implementation. To mitigate the need for excessive experiments that screen bio-related interactions through conventional pathways, *in silico* methodologies have been established as cost and time-efficient alternatives. This study presents the development of data-driven workflows for the risk assessment of ICNP-induced cytotoxicity. Two modelling strategies were established: an evidence-based approach relying on experimental features and exposure conditions, and an atomistic-based approach combining attributes that describe NPs at the atomistic scale. While the former methodology struggled to meet the requirements for robust quantitative predictions, the models implemented on the enriched dataset displayed improved accuracy. The resultant Random Forest model fully adheres to the principles outlined by the OECD for the development of quantitative structure–toxicity relationship (QSTR) models. Beyond deciphering the mechanistic influence of individual features, the effect of the selected nanodescriptors was reviewed through Shapley additive values and permutation importance analyses to reveal key input characteristics that drive cell viability predictions. The produced model is disseminated as a free to use web service hosted by the Enalos CHIASMA Cloud Platform (https://www.enaloscloud.novamechanics.com/chiasma/icnp/) and data are publicly available through the NanoPharos database for easy access to the nano-safety community.

## Abstract 2

Iron carbide nanoparticles, a type of magnetic nanostructure, have been proposed as novel candidate therapeutic agents for a wide range of biomedical applications, yet their biocompatibility remains a critical concern for their safe implementation.

## Graphical abstract

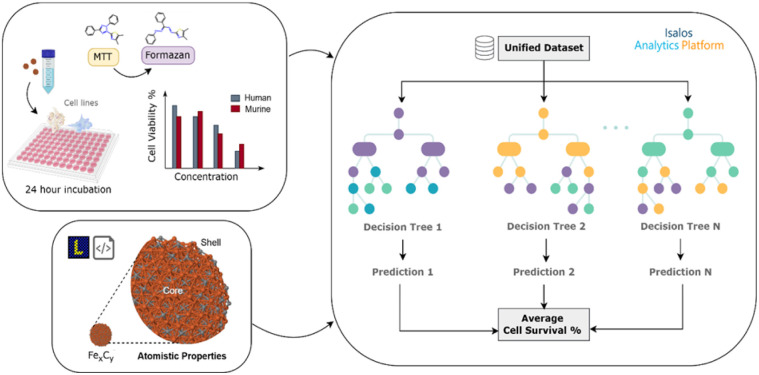

## 1 Introduction

Decades of research in nanotechnology have revolutionised numerous industries, from agriculture and cosmetics to energy storage and electronics.^1,2^ In medicine and healthcare, nanomaterials (NMs) have long been explored for their potential in diagnostics and treatment modalities.^3^ Their high surface-to-volume ratio, tuneable physicochemical properties, and ability to interact with biological systems at the molecular level have led to advances in drug and gene delivery, imaging, biosensing, and regenerative medicine.^2^ Magnetic nanoparticles (MNPs) are particularly promising due to their unique superparamagnetic behaviour and controlled manipulation by external magnetic fields.^4,5^

Iron carbides have long been recognised as active catalytic phases in Fe-based Fischer–Tropsch synthesis, where the cementite (θ-Fe_3_C), hexagonal (ε-Fe_2_C), pseudo-hexagonal (ε′-Fe_2.2_C) and Hägg carbide (χ-Fe_5_C_2_) crystalline phases are formed as intermediates.^6,7^ Beyond the bulk material's industrial relevance for liquid fuel production, iron carbide nanoparticles (ICNPs) have recently been proposed as candidates in biomedical applications.^8–10^ In bioimaging, they serve as effective contrast agents in Magnetic Resonance Imaging (MRI) and Photoacoustic Tomography (PAT). They exhibit excellent superparamagnetic properties, such as high saturation magnetisation (*M*_s_ ∼ 140 emu g^−1^), that enable them to improve MRI contrast by shortening the relaxation times of surrounding water molecules.^9,11^ Fe_5_C_2_ nanostructures were reported to outperform iron oxide nanoparticles (IONPs) in generating hypo-intensities on T_2_-weighted MRI maps due to their superior transverse relaxivity (*r*_2_) values, with ICNPs exhibiting an *r*_2_ of 464.02 mM^−1^ s^−1^ compared to 178.30 mM^−1^ s^−1^ for IONPs.^12,13^ In a therapeutic context, ICNPs have shown great promise as heating mediators for magnetic hyperthermia (MHT).^6,14,15^ Their high specific absorption rates enable efficient conversion of external energy into heat under an alternating magnetic field to target tumour tissues.^16,17^ Their potential extends further to photothermal therapy, photo- and chemo-dynamic therapy,^18^ and controlled delivery of antitumour drugs (*e.g.*, doxorubicin).^19^

ICNPs are perceived as safer alternatives to other magnetic NPs, as their carbon content improves the chemical inertness of the NP and prevents oxidation. They present higher thermal stability over metallic iron and iron oxide.^20^ Herrmann *et al.* conducted a long-term exposure study on carbon-encapsulated ICNPs in mice, revealing that despite persistent localisation in lung and liver tissues, hardly any necrosis, tumorigenesis, fibrosis, or inflammation signs were detected over a one-year period.^21^ We have recently prepared a systematic review (SR) examining the toxicity of ICNP exposure in biomedical contexts, that showed satisfactory biocompatibility of the material in an *in vitro* setting.^22^ Nevertheless, an *in silico* strategy to assess their toxicological impact is still lacking.

The cytotoxic potential of ICNPs can be examined through the development of computational methods that correlate their distinct physicochemical characteristics with their toxicity profiles. Nanomaterials have a vast array of properties that influence their interactions with the surrounding medium upon exposure to biological environments. Owing to their high surface energy they absorb biomolecules (*e.g.*, proteins, lipids and polysaccharides), which leads to the formation of a biomolecule corona, a phenomenon that modifies their identity.^23–25^ Additionally, complex interactions control mechanisms like oxidative stress, membrane disruption, and generation of reactive oxygen species (ROS), which collectively govern cytotoxic outcomes.^26^ Predicting NMs' cytotoxicity *in silico* is inherently a non-straightforward task, as it requires a model capable of capturing this complexity. Mathematical frameworks and physics-based simulations have proven instrumental in addressing this challenge; grouping and read-across models,^27^ nano-quantitative structure activity/toxicity relationships (nano-QSARs or QSTRs),^28–30^ adverse outcome pathways (AOPs)^31,32^ and physiologically based pharmacokinetic (PBPK) models^33,34^ have previously been established for a range of inorganic NMs. Such methods have gained tremendous popularity in nano-safety assessment during the past decade as an alternative to traditional *in vivo* and *in vitro* testing. The emergence of artificial intelligence (AI) and increasing availability of NM-focused databases has produced a growth in machine learning (ML)-based nano-QSARs.^35^

*In silico* studies in nanotechnology are immensely delimited by small datasets, usually with little variation in the reported NMs properties (in part driven by the relatively narrow scope of read-across in the EU regulatory context^36^ which requires a common core composition as a basis for structural similarity read-across approaches), and heavy reliance on experimental results and/or computationally intensive simulations.^37,38^ This often leads to case-by-case studies focused on specific types of NPs, and results in models with constrained applicability to other classes of NMs. Nanotoxicity studies with broader applicability prospects must undergo laborious data mining processes, given that the required information is extracted from one-at-a-time experiments. Recent efforts by Labouta *et al.*^39^ and Shirokii *et al.*^40^ have focused on collecting cytotoxicity evidence from multiple individual studies across various NM types. Meta-analyses were conducted on these large, diverse datasets, allowing them to validate their findings on external data.

Another challenge is the lack of universally accepted descriptors, which are essential for representing the distinctive characteristics of NP samples. Nanodescriptors encoding size, shape, chemical composition, surface charge are typically acquired from direct measurements after NP synthesis, through advanced microscopy techniques and other analytical methods. Beyond experimental properties, theoretical descriptors serve as supplementary input for models in nanotoxicology.^38^ Quantum–mechanical properties derived from density functional theory (DFT) methods (*e.g.*, highest occupied/lowest unoccupied molecular orbital (HOMO/LUMO) energies, enthalpy of formation, absolute electronegativity, *etc.*), as well as periodic table descriptors (*e.g.*, atomic radii, periodic number of metals, the number of valence electrons, *etc.*) have been widely used as input to nano-QSARs in the past. Other techniques have been successful in annotating the three-dimensional nanostructures, while accounting for their size, shape and attached ligands.^41,42^ Despite their high computing requirements, these full particle nanodescriptors capture geometric and topological features and give a measure of the NMs' stability under the exposure conditions. Computational descriptors from the 3D representation of inorganic NPs have been incorporated into ML pipelines in NMs' toxicology research.^43–46^

ML has proven invaluable not only in predicting NM's properties and adverse effects, but also in interpreting a model's decisions with different explainable AI (XAI) techniques. Since most ML models are designed for use and interpretation by non-informatics experts (*e.g.*, experimentalists, industry stakeholders, regulators and risk assessors), it is essential to strengthen user confidence in the model's utility by explaining the factors that drive a prediction. XAI's goal is to understand the reasoning behind the generated predictions, rather than just the outcomes themselves.^47^ The inclusion of mechanistic insights from omics data and AOPs into ML models is also expected to open up the opaque nature of apical endpoints currently used in much of toxicological regulation which give very limited insights into the source of any observed effects *in vivo*.^48^ Yu *et al.* showcased the advantages of XAI in nanoinformatics through two studies: one on predicting the toxicity of metal oxide and quantum dot NPs,^49^ and another on investigating NP uptake during seed priming.^50^

This work compares two *in silico* methodologies to examine the biological response of ICNPs in a physiological environment. The initial modelling procedure involved using the current knowledge gathered from the previously conducted SR.^22^ The modelling procedure was further amplified by atom-level structural information derived from the crystal structure of the bulk iron carbide material. By comparing the predictive accuracy of these methods, we demonstrate the added value of integrating structural NP information for improved predictive performance. Furthermore, model interpretability techniques were pursued for both physicochemical and structural features supporting users in understanding the basis on which the model predictions are generated. The curated dataset has been made available in a dedicated database, and the final trained model has been deployed as a web service for easy access.

## 2 Materials and methods

### 2.1. Dataset

In our previous work, information on the toxicological profiles of ICNPs applied in a biomedical context was collected *via* data mining the literature, with the majority of the included studies using colorimetric assays to evaluate the biosafety of ICNPs. Ten immortalised cell lines from human and murine organisms were exposed to the NPs, and the percentage of surviving cells compared to a control sample was measured at different NP concentrations. All viability endpoints (*n* = 186 data points) correspond to a 24-hour treatment period, which represents the dominant timeframe in the conducted studies. Shorter and longer incubation times were excluded due to paucity of available data, as these time points did not provide sufficient coverage of ICNP chemistries.

The unified dataset comprised nine attributes related to physicochemical properties, the experimental setup and exposure-related factors. The diameter of the spherical iron carbide cores (ranging from 4.9 to 44.3 nm) and the thickness of the shell material, which was mostly around 2–2.5 nm, were also extracted from the publications. Each experimental sample has a distribution of NP sizes, serving as a measure of size uniformity. The standard deviation from the average size in each treatment was derived from the studies to quantify the variability in size, as narrower NP size deviations are often associated with more uniform cellular interactions and internalisation rates.^51,52^

**Fig. 1:**
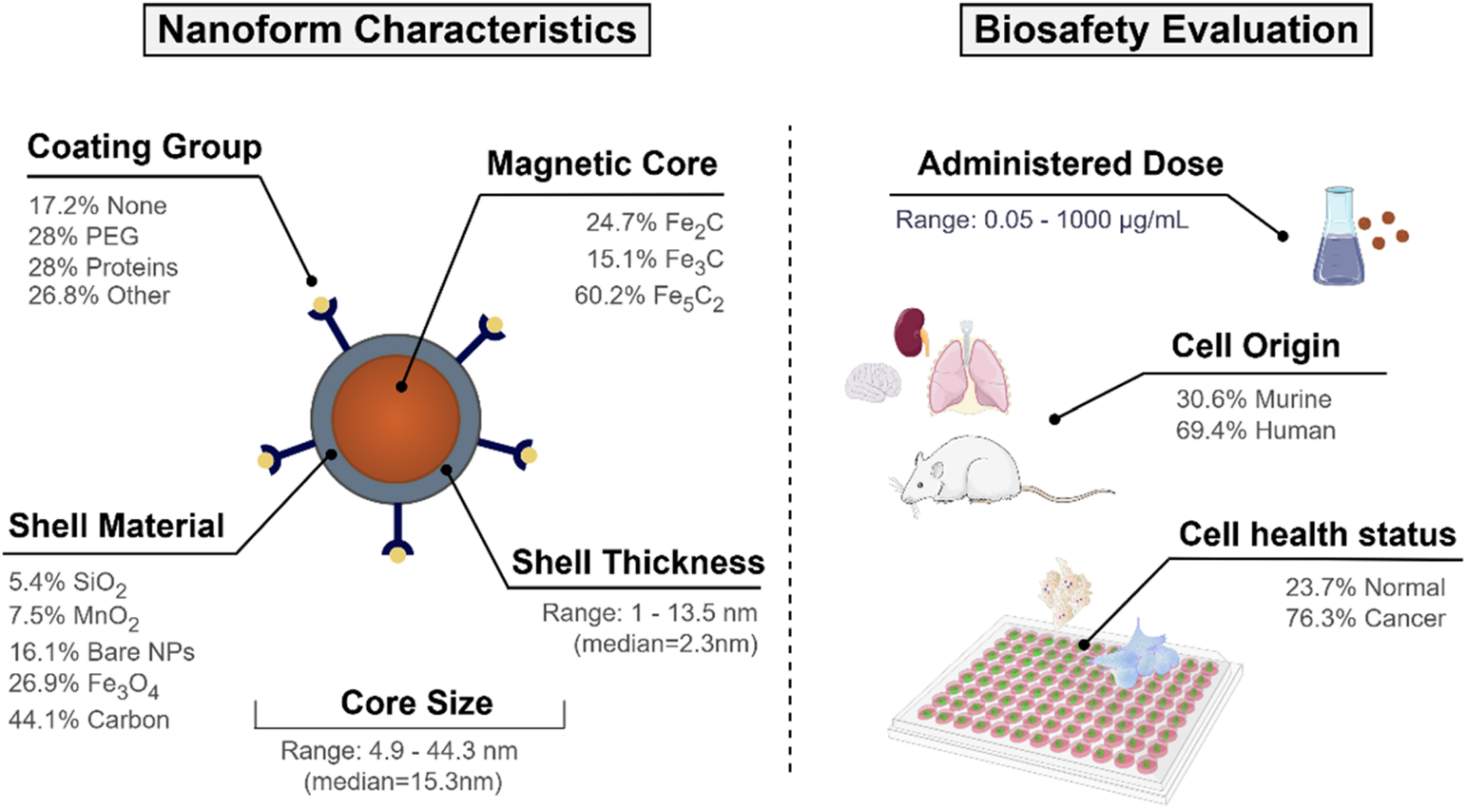
Schematic representation of the experimental design of ICNPs, including the distribution of core phases, shell materials, surface modifications, size ranges, and exposure concentrations. The dataset includes both murine and human cell models used in cytotoxicity assessments.

In addition, information about the surface chemistry of ICNPs was gathered. Where present, shell materials encircling the iron carbide core included layers of iron oxide, amorphous carbon, silica or manganese dioxide. Surface modifications with therapeutic molecules, stabilising agents and stealth coatings were also documented. Different chemical moieties, such as poly(acrylic) acid,^53^ pluronic acid,^15^ and polyethylene glycol (PEG),^18,54^ were used for surface alterations to amplify the particles' biocompatibility. Lastly, the remaining attributes were related to the experimental setup, including the conditions under which the biological evaluation took place (*i.e.*, cell type, treated organism, cell line health status (normal or cancer cells)). A graphical summary of the experimental dataset composition is presented in Fig. 1.

### 2.2. Atomistic descriptors for data enrichment

Nanodescriptors that characterise the NPs at the atomic scale were generated using a modified version of the NanoConstruct toolbox.^55^ The ICNP spherical cores of different sizes found in the main dataset were constructed from the unit cell of the material. NP structures were derived from the most thermodynamically stable crystal configurations of the respective iron carbide phases, and they correspond to energy-minimised formations of monodisperse, uncoated NPs in vacuum. A core–shell model was applied to describe the structural composition of the NPs, where the shell region was defined as the atomic layer extending up to 4 Å from the surface, while the remaining atoms formed the NP core. This approach facilitated the derivation of nanodescriptors quantifying essential features of surface and bulk atoms. The tool calculates atomistic descriptors for three NP regions: the entire particle, the iron carbide core and the shell region surrounding the core.

The Crystallographic Information Files (CIF) of three iron carbide phases were derived from the Crystallography Open Database (Fe_2_C: 1543664.cif,^97^ Fe_3_C: 1008725.cif,^98^ Fe_5_C_2_: 1521831.cif^99^). The NanoConstruct tool generates a list of candidate force fields from the OpenKIM repository,^56^ ranked from less to more generic based on the chemical elements present in the respective CIFs. For Fe–C phases, we selected the modified embedded atom method (MEAM) interatomic potential by Liyanage *et al.*.^57^ The MEAM parameters have demonstrated good agreement with DFT results and experimental measurements (±5%) for bulk iron and Fe–C alloys^58^ and have been benchmarked for nanoscale iron carbide particles of ∼5 nm in size.^59^

A total of 57 atomistic descriptors related to the macroscopic structure and microscopic properties of the NPs were computed to augment the main dataset. The computed descriptors included, but were not limited to, the number of atoms present, NP volume and surface area, average potential and lattice energies and force-related descriptors (*e.g.*, coordination parameters at cutoff distances of 3 Å, 4 Å, and 5 Å). These descriptors were calculated either for the three mentioned regions, or as descriptors expressing ratios or differences between core and surface atoms. More information on the comprehensive list of the computational descriptors can be found in the original publication describing NanoConstruct.

### 2.3. Overview of the model development process

**Fig. 2:**
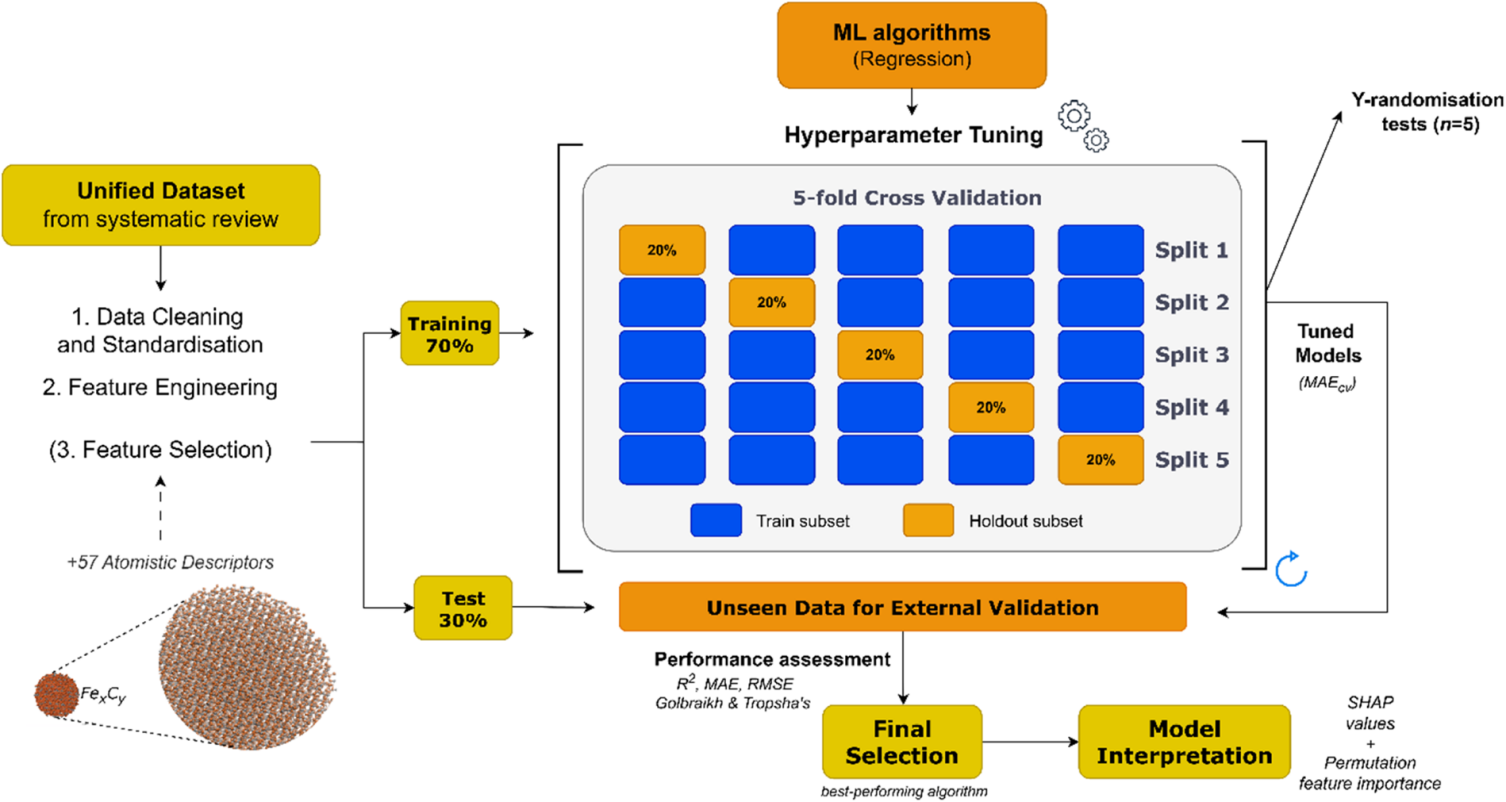
Model development schema: 70% of the assembled dataset was used to train and build models from a pool of ML algorithms. A nested *k*-fold (*k* = 5) cross validation technique was used for hyperparameter optimisation, where one fifth of the instances were used for validation in each split. The remaining 30% of the data (blind set) was used for external validation, which was followed by model interpretation.

The current work examines two approaches to predict possible cytotoxicity caused by ICNPs. The first relies solely on the evidence extracted from the literature to model cell viability in a quantitative manner. The second approach estimates the cell survival percentile by combining atomistic-based information that characterises the NP structures (Section 2.2) with the existing experimental knowledge. The general proposed workflow followed for model development in both cases is depicted in Fig. 2. The overall workflow was conducted using the Isalos (https://isalos.novamechanics.com/) (ref. 60) (v.0.4.0) analytics platform, and the Python programming language.

The following algorithms were examined in an iterative process: Random Forest (RF), *k* nearest neighbour (kNN), Linear Stochastic Gradient Descent (SGD), Multi-layer Perceptron (MLP), Light Gradient-Boosting Machine (LightGBM), and XGBoost Regressor. They represent diverse learning paradigms, including ensemble-based methods (RF, XGBoost), distance-based proximity models (kNN), linear regression approaches (Linear SGD), and neural network architectures (MLP). They were fitted on a portion of the initial data designated for training (70%), which was separated from a test set for unbiased evaluation.

To optimise the performance of each surveyed algorithm, fine-tuning was conducted with a randomised search using parameter combinations from a specified grid. An inner loop was used for hyperparameter selection and an outer loop for external model validation (Fig. 2). In the inner loop, a nested *k*-fold (*k* = 5) cross-validation (CV) procedure was used to train each model multiple times using a distinctive subset as the training set and the remaining 20% subset for validating purposes.^61^ In the nested CV scheme, the performance of the trained models for each training fold was evaluated with Mean Absolute Error (MAE_cv_) and the highest score averaged over the five holdout folds determined the best-performing algorithm for each task. A summary of the key hyperparameters optimised for each algorithm is provided in Table 1.

**Table 1:** Optimised hyperparameters and normalisation requirements for each machine learning algorithm

Algorithm	Optimised hyperparameters	Feature scaling
Random Forest regressor	Number of trees in the forest, tree depth, minimum impurity reduction required for a node to split	No
k-Nearest Neighbours	Number of neighbours, weight function	Yes
Linear SGD	Learning rate, maximum number of iterations	Yes
MLP regressor	Activation function for the hidden layer, initial learning rate	No
LightGBM	Number of boosted trees, learning rate	No
XGBoost regressor	Learning rate, maximum depth per tree	No

#### 2.3.1. Data preprocessing

Once assembled into a standardised dataset, preprocessing steps included addressing missing features that arose due to inconsistent reporting across individual studies. Zeta potential, a property that approximates NP surface charges and mediates cytotoxicity, was often not measured or reported in the collected studies, thus it was omitted from the analysis. Categorical attributes were converted into numerical forms with ordinal or binary encoding where necessary, to map relationships within and between features. Varied surface modifiers across the dataset were grouped according to their intended function (*i.e.*, polymer-based, protein-based, bare NPs and other).

Based on ISO standards for the performance of *in vitro* assays, a decrease in cell viability to less than 70% of the control sample is indicative of cytotoxic potential.^62^ Among the samples, 18.3% are considered cytotoxic and 81.7% non-cytotoxic. This skew can bias the model towards stronger performance in predicting non-cytotoxic outcomes. Although the modelled endpoint is a continuous measure rather than a binary toxicity outcome, stratified sampling was applied during the dataset split to maintain a balanced representation of each viability category. The dataset was split into training and test sets in a 70:30 proportion, where at least one treatment from each original experiment was retained in the hold-out set to ensure adequate representation of all available chemistries in both subsets.

Distance-based and linear supervised algorithms are sensitive to feature ranges and require normalisation, while rule-based algorithms are considered scale-invariant. When standardisation was required (Table 1), values were transformed with a *z*-score (Gaussian) function into features of zero mean value and unit standard deviation. Normalisation was determined based on the training subset and then it was applied to the test set.

#### 2.3.2. Feature selection

For the approach relying merely on experimental and physicochemical measurements, all available features were retained to capture the maximum possible knowledge from this relatively limited dataset. In contrast, feature selection was conducted for the augmented dataset due to the significantly larger pool of variables. Initially, features that only described the biological evaluation (*e.g.*, cell type, health status of the cell) were omitted, while attributes characterising the shell and coating material, the core and overall size and the exposure dose were included. The majority of calculated atomistic descriptors are dependent on nanoparticle size, introducing unwanted multicollinearity in the extended dataset. As a measure to reduce redundancy among data, attributes that contributed limited information (*i.e.*, number of atoms on core and surface) were excluded through zero-variance filtering and a subsequent pairwise Pearson correlation analysis (correlation >0.99). Five descriptors for coordination parameters at the smallest cutoff distance returned zero values and were excluded. This is observed due to the cutoff distance of 1.2 times the sum of atomic radii of Fe–C atoms being shorter than the bond lengths between Fe–C atoms in the respective unit cells, thus no neighbouring atoms are detected.

To further refine the selection, global feature importance was determined by calculating Shapley Additive exPlanations (SHAP) values for each feature (Section 2.4). SHAP-based feature selection operates by computing values for each instance, aggregating them to derive absolute mean scores for each atomistic feature and retaining the highest-ranked ones.^63^ A Random Forest Regressor with 40 estimators and a maximum depth of 10 was trained on the full feature set and a TreeExplainer^64^ was built on the established model. Attribute selection was performed on the training data, to avoid information leakage from the unseen test data.

#### 2.3.3. Performance assessment

Following the hyperparameter optimisation for a selection of ML algorithms in the nested CV schema, their performance was evaluated on the remaining portion of the initial data that was not involved in the training process (Fig. 2). The performance of the tuned models was evaluated on previously unseen data samples by quantifying the extent to which the predicted values are different to the true measured observations. The equations for all statistical metrics are provided in Table S1 of the SI for reference. The algorithm exhibiting the best overall validation scores was selected for final modelling in both the evidence-based and atomistic-based approaches.

To further assess the final model, the leave-one-out (LOO) CV technique was used due to its suitability for small datasets. To eliminate concerns about potential overfitting, the Y-scrambling technique was applied with five randomisations, to examine whether the selected algorithm learns true correlations among data rather than fitting noise. As an external validation step, the final model was evaluated according to the acceptability criteria proposed by Golbraikh and Tropsha.^65,66^ These guidelines assume a sufficient QSAR if the following conditions are met: (i) the LOO cross-validated correlation coefficient (*Q*_loo_^2^) must be greater than 0.5; (ii) the coefficient of determination for predictions (*R*^2^) must exceed 0.6; (iii) the difference between the predicted and observed determination coefficients (*R*^2^ – *R*_o_^2^) should be less than 0.1; and (iv) the slopes (*k* and *k*′) of regression lines through the origin must fall between 0.85 and 1.15.

#### 2.3.4. Applicability domain definition

The small datasets and minimal NP diversity in NMs' *in silico* studies constraints their practicality within the boundaries of the conditions used to train a model. Although defining the applicability domain (APD) in nanotoxicity modelling helps identifying the region of the input space where the model's assumptions are regarded as correct.^67^ A broad APD strategy for QSARs has yet to be adopted by modellers and regulatory authorities.^68^

A distance-based method was utilised here to define the APD, relying on the idea that the model's assumptions are more likely to be valid for data points similar to the training data, based on the proximity among those observations. Firstly, the distances between all training samples are calculated, and the subset of distances lower than their average is retained. Next, the new average value <*d*> and standard deviation of the remaining distances are determined to form an APD threshold as follows:

1 APD_limit_ = <*d*> + *Zσ*

A prediction for a query NP is deemed unreliable if the computed distance from its nearest neighbour in the training subset exceeds the predefined APD limit. We considered a dual distance assessment for defining the APD. Firstly, Euclidean distances were used, with categorical attributes (shell material and functionalised groups) excluded from the domain calculation. Subsequently, we employed the same methodology using Gower distances to quantify similarity for mixed data types. The Gower distance metric considers both continuous and categorical variables and allows feature-specific weights to be assigned according to feature importance.^69^ While this distance metric has been previously proposed as a quantitative measure of similarity in materials informatics applications,^70^ to the best of our knowledge it has yet to be introduced in an APD definition. Full details on the calculation of Gower distances and feature weights, are provided in SI (S2).

### 2.4. Explainable AI prospects

Model interpretability is particularly important in fields like nanotoxicology and risk assessment, where stakeholders' trust in computational models is still developing. Thus, toolkits with exploratory functionalities and visualisation features are used to provide iconographic aid and ensure that the underlying mechanisms governing the predictions are well-understood. Several XAI techniques such as permutation feature importance, individual conditional expectation (ICE) with partial dependence (PDP) plots, and Shapley additive explanations (SHAP) have been used in nanoinformatics research thus far. These methods can be performed either at a global (for the model as a whole) or local (for each observation) level.

SHAP is a model-agnostic method adopted from game theory that quantifies the contribution of each feature to a model's output.^71^ Formally, it provides a means for assigning a “contribution” score to each feature, which represents the impact of a feature on the predicted outcome. The SHAP value is thus the average contribution of feature *i* across all possible combinations of features, and features with higher mean absolute SHAP values are considered more contributory a model's output (eqn (S1)). Negative values were associated with parameters that drove the model's predictions towards cytotoxicity (lower cell viabilities), while positive values pushed the predictions towards non-cytotoxicity (higher cell viabilities). For this analysis, SHAP was employed both at a global level to guide feature selection, and at the local level to explain how individual features' values affect the outcome of specific observations.

Additionally, ICE and PDP plots were further used to decipher predictions. A PDP estimates the marginal effect of a given independent variable on the predicted outcomes by averaging over the influence of all other variables.^72^ ICE plots extend PDPs by visualising the individual dependence of each observation separately, resulting in one curve per instance rather than averaging effects.^71,73^ Plots for the most relevant features impacting cytotoxicity were generated using the PDPbox toolbox in Python (https://github.com/SauceCat/PDPbox/).^74^

## 3 Results and discussion

The objective of the present study is the development of predictive models for the assessment of *in vitro* cytotoxicity caused by exposure to ICNPs. Two workflows were examined for modelling the dose–response dataset reduction of cell viability after exposing immortalised cell lines to ICNPs. The former direction relied on the experimental conditions and physicochemical parameters measured after the synthesis of the NPs, sourced from independent studies. The second approach involved augmenting the original set of variables with computational descriptors that characterised the structural properties of the NP core material.

Both methodologies for nanotoxicity prediction were developed within a coherent modelling and validation framework that adhered to regulatory standards. The principles for QSAR models published by the Organisation for Economic Co-operation and Development (OECD) (updated in 2014) were followed to ensure compliance with regulations. These guidelines include: (1) a definition of the modelled endpoint, (2) the use of an unambiguous algorithm, (3) a transparent description of the model's APD, (4) appropriate methods to measure goodness-of-fit, robustness, and predictive performance of the model and (5) where feasible, mechanistic insights into the selected variables.^75^ This section presents the evaluation of the ML-based QSTRs developed for both approaches, highlights key atomistic descriptors contributing to prediction accuracy, and demonstrates the use of XAI for model interpretability. Finally, the dissemination of the finalised modelling workflow as a web application is discussed.

### 3.1. Evidence-based approach

The initial approach leveraged the maximal available knowledge derived from twelve experimental studies, containing physicochemical parameters and exposure conditions. Core diameter, shell thickness, NP concentration, surface modifications and tested cell lines were among the modelled features. Model evaluation relied on a diverse set of metrics (Table S1) and model acceptance was contingent on meeting Globraikh and Tropsha's criteria for QSARs.^65,66^

XGBoost emerged as the best performing regressor by displaying the lowest MAEcv value over the five holdout validation sets in CV (MAE_cv_ = 0.067). However, evaluation metrics applied on the two subsets (Table 2) demonstrate insufficient predictive accuracy and overfitting on the training data. Also, the model only partially meets the acceptability criteria, reflecting poor external predictivity and overfitting tendencies (Table 3). None of the six tested algorithms meet all of the validation criteria, which underscores the challenge of developing fully acceptable QSTRs using low-sample datasets. In the case of ICNPs, algorithms trained on the initial dataset alone are not capable of capturing more complex relationships and extrapolating to quantitative predictions, justifying our strategy to augment the original data collected from the SR.

**Table 2:** Model validation metrics based on test and training subsets for the evidence-based approach

Statistical measure	Test set	Training set
*R* ^2^	63.5%	95.2%
Adjusted *R*^2^	56.4%	94.8%
Mean absolute error	0.089	0.014
Mean squared error	0.021	0.002
Root mean squared error	0.146	0.045

**Table 3:** Golbraikh and Tropsha's acceptability criteria for the XGBoost Regressor

Metric threshold	Score	Acceptability criteria met
*R* ^2^ > 0.6	*R* ^2^ = 0.635	Yes
*Q*_loo_^2^ > 0.5	*Q*_loo_^2^ = 0.317	No
*r* ^2^ − *R*_0_^2^/*r*^2^ < 0.1	*r* ^2^ − *R*_0_^2^/*r*^2^ = 0.518	No
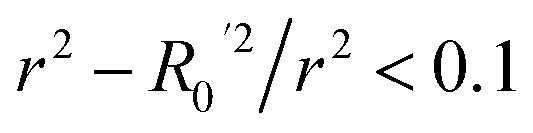	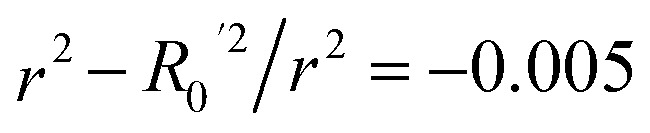	Yes
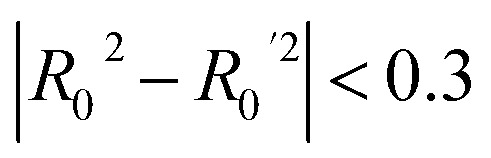	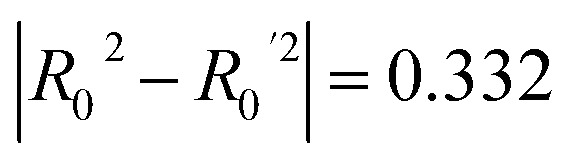	No
0.85 < *k* < 1.15	*k* = 0.983	Yes
0.85 < *k*′ < 1.15	*k*′ = 0.988	Yes

### 3.2. Atom-based approach

Subsequently, we incorporated atom-level structural descriptors as additional features. Initially, 57 computational descriptors were extracted to characterise the NPs of different iron carbide phases and core sizes. A filter was applied on the supplemented set of features to exclude the highly correlated features, narrowing the pool to 33 descriptors (Fig. S1). The attributes removed by the correlation filter were mainly related to the atomic count distribution within the NP core and surface, or they involved atomic arrangement properties such as bond orientation and surface density.

After feature reduction, SHAP analysis was conducted on the training subset to evaluate the global importance of each atom-level attribute. The eight most contributing atomistic descriptors were prioritised, as listed in Table 4. Five variables from the initial dataset (core size, surface chemistry, shell material, shell thickness, and administered dose) that are typically known prior to NP synthesis were also selected, resulting in a final set of 13 key descriptors.

**Table 4:** Global feature importance sorted by mean SHAP value for the top eight influential atomistic descriptors

Notation	Selected descriptor	Mean SHAP value
D46	The average second hexatic order parameter of all atoms	0.0163
D7	The average difference of the potential energy between core and shell atoms (eV)	0.0137
D32	Lattice energy of NP (eV)	0.0129
D5	The average potential energy of the core atoms (eV)	0.0126
D15	The average coordination parameter (3 Å) of the core atoms	0.0112
D48	The average second hexatic order parameter of the shell atoms	0.0077
D21	The average coordination parameter (4 Å) of the shell atoms	0.0072
D33	Lattice energy of bulk material – lattice energy of NP (eV)	0.0061

Model evaluation was initiated with the nested CV on the training subset, with RF achieving the lowest CV error of MAE_cv_ = 0.026. Performance metrics on both training and test subsets are listed in Table 5, with the best-performing algorithm highlighted in bold. The RF model's generalisation capability is evidenced by consistent performance metrics between the two subsets. The evaluation against Golbraikh and Tropsha's acceptability criteria (Table 3) is as follows:

**Table 5:** Training and test set metrics for each selected algorithm

	Subset	*R* ^2^	Adjusted *R*^2^	MAE	MSE	RMSE
**Random forest**	**Test**	**0.844**	**0.704**	**0.063**	**0.007**	**0.083**
	**Training**	**0.888**	**0.859**	**0.044**	**0.005**	**0.074**
k-Nearest neighbours	Test	0.518	0.087	0.109	0.021	0.146
	Training	0.551	0.438	0.101	0.022	0.148
Linear SGD	Test	0.251	−0.420	0.125	0.033	0.182
	Training	0.310	0.136	0.125	0.034	0.184
MLP regressor	Test	0.071	−0.760	0.136	0.041	0.203
	Training	0.325	0.155	0.124	0.033	0.182
LightGBM	Test	0.257	−0.410	0.125	0.033	0.181
	Training	0.258	0.071	0.131	0.036	0.191
XGBoost regressor	Test	0.807	0.635	0.061	0.009	0.092
	Training	0.924	0.905	0.021	0.004	0.061

• *R*^2^ = 0.844 (Pass).

• *Q*_loo_^2^ = 0.639 (Pass).

• *r*^2^ − *R*_0_^2^/*r*^2^ = 0.082 (Pass).

• 
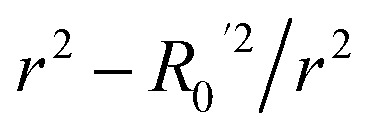
 = −0.001 (Pass).

• 
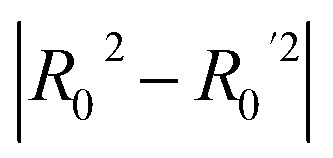
 = 0.068 (Pass).

• *k* = 0.994 (Pass).

• *k*′ = 0.995 (Pass).

**Fig. 3:**
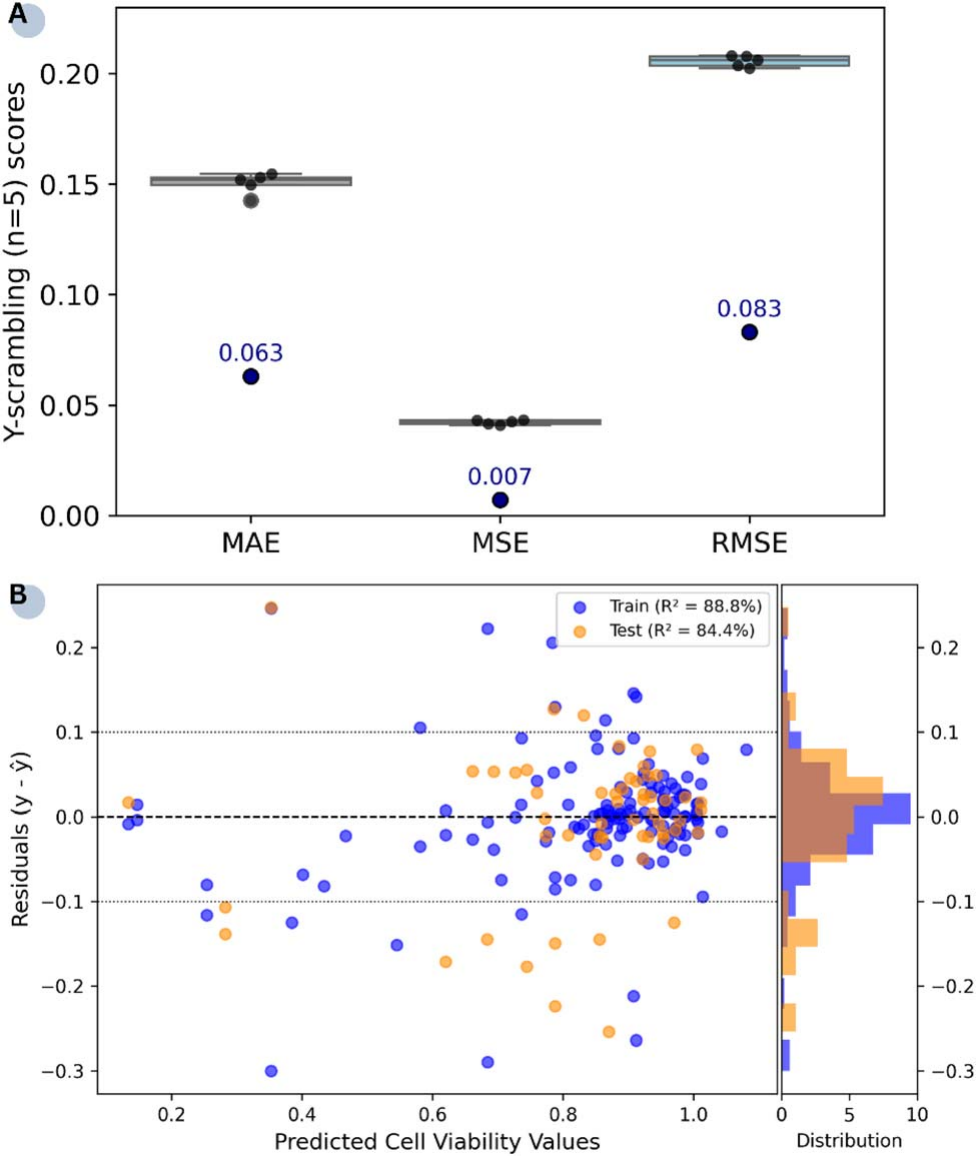
Performance of the RF model: (A) validation results over five *y*-randomisations. The blue dots represent the external validation metrics (test set) for clarity, and (B) residual plots between the true (*y*) and predicted (*ŷ*) cell viability values with ±10% tolerance lines.

All requirements for external predictivity are met. Results from the Y-scrambling test (Fig. 3A) show that RF outperforms the models trained on the scrambled response variable, whose error metrics are clustered at higher values. This outcome demonstrates that the selected model captures genuine correlations within the data rather than relying on chance patterns. The residual plot (Fig. 3B) illustrates the difference between true and predicted cell viability values for the training (*R*^2^ = 88.8%) and test (*R*^2^ = 84.4%) sets. Apart from a few outliers in both subsets, the residuals are clustered relatively narrowly around the zero line. However, the relative sparsity of data points in the lower cell viability region (<40%) may affect the model's effectiveness in detecting highly toxic conditions.

Next, a qualitative measure of model uncertainty was specified by defining the APD, according to OECD recommendations (principle (3)).^84^ The obtained APD threshold value was equal to 96.307 for Euclidean distances and 0.2652 for Gower distances, with all test samples falling within the APD limits. All key information of the final model is fully documented *via* a QSAR Model Reporting Format (QMRF) template (S3 in the SI) for transparency.^76^

Noticeably, the effectiveness of the tuned regression algorithms applied on the extended set of features improves vastly, compared to using the initial experimental data alone. The atomistic-based approach yields significantly lower cross validation scores (MAE_cv_ = 0.026–0.044) across all tested algorithms (Table S2). Full grid search settings and hyperparameter configurations for the two methodologies are provided in Table S3 of the SI file accompanying this work.

### 3.3. Limitations of the proposed methodology

While the current approach shows significant improvement in predicting ICNP-induced cell viability, several limitations must be acknowledged. A key consideration is the lack of experimentally measured properties such as zeta potential and hydrodynamic size. These parameters are used as markers of surface charge and stability/accessibility to cells, respectively, and are known to influence the interactions of NPs with biological systems.^77,78^

Substantial limitations originate from the data used in this study, as the dataset reflects exposure at a fixed time duration. This incubation time may not capture meaningful cytotoxic effects over longer exposure scenarios, as prolonged exposure causes toxicity mechanisms different from those observed at a 24-hour time point.^79^ The model is therefore calibrated for a single-time effect, and extrapolation to shorter or longer exposures falls outside its applicability domain.

The developed models were trained and validated on aggregated data with high heterogeneity in terms of NP characteristics and exposure conditions. For instance, conjugated ligands and NP concentrations were selected independently across the different studies each of which were performed in different labs by different research groups. The presence of a few outliers in Fig. 3B underscores the need for further data collection to capture the full spectrum of cell survival responses. In addition, since no oversampling methods were applied to avoid synthetic redundancy, subtoxic treatments are being overrepresented in the dataset. The limited dataset size is reflective of the poor availability of nano-related data in the public domain. The scarcity of data and class imbalance remain limiting factors for model generalisability.

Another potential weakness of the current approach is that extrapolation of *in vitro* findings to *in vivo* scenarios is not feasible. The present work assumes that NMs adjacent to the ones found in the dataset induce similar cytotoxic effects. However, the response of living organisms to the ICNPs remains speculative.^80,81^ Finally, the model covers only the metabolic activity of immortalised cells after NP exposure as a cytotoxicity endpoint. Other critical biochemical metrics including cell uptake, protein expression, ROS production, and cell death, which may portray a NMs' biosafety profile more thoroughly, were not taken into account due to inadequate reporting in the limited number of available studies. This may be due to the focus on IONPs for use in biomedical applications – a widening of the search criteria to toxicological studies more generally may result in complementary data. As the models were built using data strictly for Fe_*x*_C_*y*_ spherical structures, the extrapolation to other types and shapes of magnetic nanostructures should be carefully considered by the users.

### 3.4. Interpretation of the selected descriptors

The intent of the fifth OECD principle for QSAR model development and validation is to ensure that there is an association between the descriptors used and the endpoint predicted; if a descriptor in the model is considered as important, that means it may act as a decisive factor in the cell viability reduction caused by the NMs.

At first, the selection of concentration is plausible, considering that the data used for modelling are derived from dose-dependent viability plots. Moreover, NP toxicity is believed to be influenced by their size, since it controls their surface-to-volume ratio and directly affects the translocation, diffusion, and accumulation of NPs in different organs and cell tissues.^26^ Shell materials and coating groups surrounding the NPs were selected owing to their influence on nano-bio interactions. The surface functional groups determine how ICNPs interact with cell membranes and tissues, shaping the protein corona formed from the cell culture medium serum and ultimately impacting cytotoxicity.^82,83^

The use of atomistic computations allows the transition from the macroscopic to the microscopic level in the assessment of the influence of NP properties on the toxicity they induce in cells. The average potential energy of core atoms and the potential energy difference between core and shell atoms were selected due to their correlation to the NP's structural stability. Lower potential energies are indicative of more stable structures, while large differences between the two layers of atoms reflect potential instabilities. The lattice energy of a NP and its difference from that of the bulk material's lattice energy are also descriptive of the particles' stability. Coordination parameters, at a cutoff distance of 3 Å (D15) for core and 4 Å for shell (D21), provide information into NP's atomic arrangements by calculating the average number of neighbouring atoms of a single atom.^84^

**Fig. 4:**
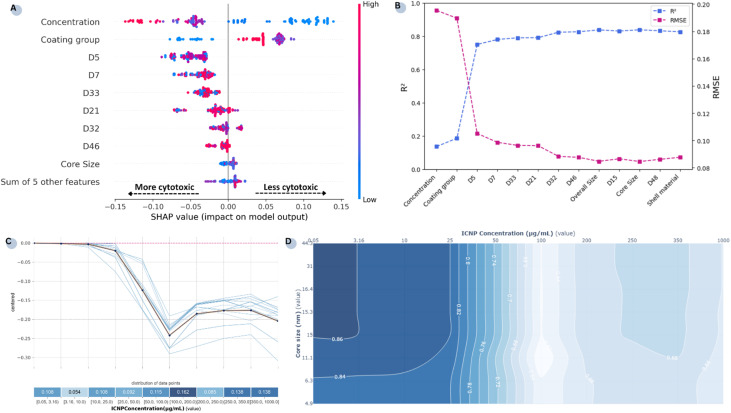
(A) Local interpretability summary for the selected descriptors. (B) *R*^2^ and RMSE evolution with progressive addition of features to a RF model (attributes are ranked by average SHAP value). (C) PDP (highlighted in orange) and ICE plots (blue lines) for the most relevant feature correlating cell viability in the RF model. The feature distribution is displayed in bins below the *x*-axis, and the *y*-axis shows the centred effect of concentration on the predicted outcome. (D) Two dimensional PDP between ICNP concentration and the core size of the NPs. Darker blue regions indicate higher predicted viability.

In addition to interpreting the influence of individual features mechanistically, XAI techniques were employed to decipher the model's decision-making process. A *post hoc* SHAP analysis was used to describe how each NP property influences the model's outputs in understandable terms. The summary plot in Fig. 4A shows the magnitude of each feature's impact on the model's final cell viability/cytotoxicity predictions when compared to the average prediction. Each dot corresponds to a sample of ICNP cellular toxicity obtained from the entire extended dataset, and the colour signifies the feature's value. The features are ranked by greater importance and those exhibiting negative SHAP values drive the model's output towards lower survival percentages (*i.e.*, higher cytotoxic effects).

The results indicate that experimentally derived parameters contribute more to the final prediction, with the features ‘concentration’ and ‘coating group’ identified as the most determinant ones. These findings are in agreement with the subgroup analyses conducted in our previous work.^22^Fig. 4B illustrates how the predictive performance of the RF model evolves as features are incrementally added. While concentration and coating groups have emerged as the most influential factors driving cytotoxicity during the previous analysis, it is clear that these two features alone are insufficient in achieving reliable predictions and that performance improves considerably with the inclusion of computational attributes. Notably, although individual atomistic-level features appear to have a limited direct effect on the response regardless of their magnitudes, their combined presence in the model is paramount.

More precisely, higher concentration values and coating groups of lower ordinal values (encoding uncoated NPs) typically drive the output towards reduced cell survival rates. As shown in the isolated PDP (Fig. 4C), at very low concentrations predictions near zero effect and at doses higher than 10 µg mL^−1^ ICNPs have a noticeable negative impact on cell survival. Reduction in cell viability with increasing dose persists up to 100 µg mL^−1^ and further increases beyond that point have a small additional effect. Also, while smaller ICNP core sizes were associated with higher cytotoxicity in Fig. 4A, the direct impact of this feature on model predictions is minimal. This suggests that NP size alone does not influence outcomes in comparison to dose and surface modifications. The trivial effect of ICNP core size is also affirmed by the feature's interaction with the most determinant feature (Fig. 4D), where NP size impacts toxicity only at low doses (<25 µg mL^−1^). At higher doses, the size becomes secondary as concentration fully drives cytotoxicity.

### 3.5. Web implementation *via* the Enalos CHIASMA Cloud platform

**Fig. 5:**
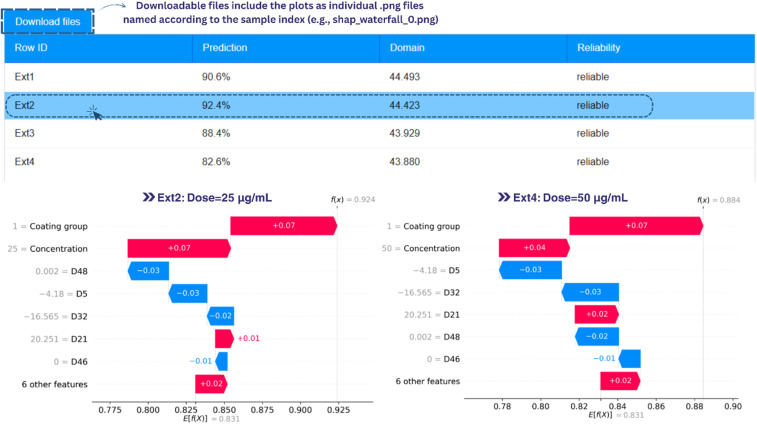
Example output from the web service. The results for each submitted sample include the cell viability prediction, an indication of reliability based on APD with euclidean distances, and waterfall plots by clicking on a sample of interest. Features contributing to increased and decreased predicted viability appear in red and blue, respectively. The two plots correspond to two datapoints from the blind set from ref. 93.

In an attempt to facilitate the safety assessment of ICNPs, the RF model was integrated as an online web application *via* the Enalos CHIASMA Cloud Platform. A collection of computational and informatics workflows on drug discovery,^85,86^ chemical safety estimation,^45,46,87^ property calculation^88,89^ and virtual nanostructure construction^55,90^ are hosted on the Platform. Their distribution as freely offered Graphical User Interfaces (GUIs) makes these models Findable, Accessible, Interoperable, Re-useable (FAIR), and benefits domain experts unfamiliar with programming and ML trends. The web tool developed for this study can be easily accessed through the link https://www.enaloscloud.novamechanics.com/chiasma/icnp/ or *via* an application programming interface (API) for remote access, allowing fast evaluation of ICNP toxicological impact. Besides numerical estimations, the web service returns interpretability plots for each submitted sample that illustrate the marginal contribution of each feature value, helping users understand why certain nano-formulations are estimated as potentially hazardous (Fig. 5).

As noted above, limited input data (size, dose and surface functionalisation) are enough to generate a reliable cell viability prediction for ICNPs. Interested users can utilise the NanoConstruct software to derive the eight atomistic descriptors required as input (see Table 4), by simply specifying the crystal phase (*e.g.*, CIF) and size of the material. The tool supports a virtual screening environment: researchers are able to introduce multiple entries for different input parameters and observe how different ICNP characteristics might elicit undesirable cellular responses.^87^ Virtual screening allows unfit and unfavourable candidate materials to be discarded before allocating time and resources for their synthesis. Instead, experts can prioritise and design potential NMs most likely to be safe for humans and animals and that possess the desired properties for example of cellular uptake and retention for imaging, or payload capacity for drug delivery applications.^91,92^ In this context, the developed service can aid in reducing the number of NP syntheses and complementary experiments needed to test their biocompatibility.

To demonstrate the applicability of our methodology on a real-world scenario, we applied it to an blind dataset obtained from Castellano-Soria *et al.*,^93^ which was not used in the original training or test sets. This study evaluated the biocompatibility of medium-sized Fe_3_C@C core–shell ICNPs (Fe_3_C phase: *Pnma*62, graphite shell coating of ∼2.5 nm in thickness) using the same experimental protocol adopted in our training data (24 hours *in vitro* exposure on MCF-7 tumour cells), on administered doses between 12.5 and 100 µg mL^−1^. Despite the small sample size (*n* = 4), the predicted values showed good alignment with experimental observations, achieving a MAE_ext_ of 0.033. To quantify uncertainty, a bootstrapping procedure was applied, in which the initial dataset was resampled with replacement 1000 times, and the RF model was refitted for each bootstrap sample. Validation metrics and 95% confidence intervals generated for the out-of-bag data are reported in the QMRF (Section S3 in SI). Fig. 5 presents the GUI output of this example, and the waterfall plots for two datapoints corresponding to concentrations of 25 and 50 µg mL^−1^. In this case, the absence of coating on the magnetic NPs appears to be the main feature pushing the output towards non-cytotoxicity.

Model-friendly nanomaterials databases (*e.g.*, eNanoMapper, S2NANO, NanoPharos, *etc.*) are fundamental in promoting data reusability and sharing for widespread use among the scientific community.^30,94,95^ In alignment with the FAIR principles,^96^ the curated dataset was uploaded to NanoPharos, a repository that hosts ready-to-use datasets for nano-QSAR development. All information, including the raw and extended data used in this study, can be accessed *via* the link https://db.nanopharos.eu/Queries/Datasets.zul?datasetID=np29 and *via* an API (https://db.nanopharos.eu/Queries/Datasets.zul?datasetID=np29) for further integration into ML/modelling platforms. Assessments for other material chemistries tested in either similar physiological contexts or using multi-timepoint assays can be used to supplement the current NanoPharos entry and support the development of an approach with broader applicability domain.

## 4 Conclusion

In this work, we implemented computational methods for the hazard assessment of iron carbide nanoparticles (ICNPs) in a physiological context, a magnetic type of nanomaterial with relatively sparse biosafety data. Two distinct approaches for the prediction of cytotoxicity were explored: (1) one relying purely on the maximal available knowledge from the experimental dataset generated through a systematic review, and (2) a hybrid one combining computational descriptors that correspond to different properties and structural characteristics of the magnetic cores on the atomistic level.

In the first approach, an XGBoost algorithm demonstrated the best predictive performance, displaying the lowest MAE_cv_ value (0.067) in a cross validation scheme. However, the model was not successful in yielding reliable quantitative predictions for cell viability, since further evaluation revealed overfitting and inadequate external predictivity. In the latter approach, five experimental features -core size, surface chemistry, shell material, shell thickness, and exposure concentration-were collated with atomistic descriptors indicative of NP stability. The RF algorithm was selected among a panel of algorithms to proceed with the rest of the analysis (MAE_cv_ = 0.026), and it was validated using external validation, cross validation, and y-scrambling methods. The optimal model trained using both experimental and atomistic descriptors, achieved an *R*^2^ of 84.4% (on the test set) and met all the acceptability criteria for quantitative predictions, outperforming the model developed with the former approach.

A mechanistic interpretation was provided, and model-agnostic methods were employed to break down the generated outputs to useful insights into the model's decision-making process. SHAP analysis revealed that even though ICNPs' size exhibits negligible association with induced toxicity in cells, other experimentally derived features – concentration and coating composition – have the greatest contribution to the model's output. Feature addition analysis showed that the supplemented atomistic descriptors, although weak predictors individually, boost the RF's performance when combined with the selected experimental inputs. The final workflow fully adheres to the principles outlined by the OECD for the development of QSARs and their documentation *via* a QMRF. A novel APD definition is presented for datasets with mixed data types, complimenting a well-established distance-based thresholding methodology to account for both numerical and categorical features.

The eventual model was disseminated as a publicly available web application with an intuitive GUI to simplify its accessibility among interested stakeholders. The web tool supports the safe-by-design strategy, since it enables virtual screening of potential ICNPs by showcasing how different NP characteristics influence cell viability.

## Author contributions

Maria Antoniou: methodology, validation, formal analysis, data curation, visualisation, writing – original draft, conceptualisation; Dimitra-Danai Varsou: writing – review & editing; Andreas Tsoumanis: software; Georgia Melagraki: conceptualisation, writing – review & editing; Iseult Lynch: writing – review & editing; Antreas Afantitis: conceptualisation, supervision, writing – review & editing, funding acquisition.

## Conflicts of interest

M. A., D.-D. V., A. T. and A. A. are affiliated with NovaMechanics Ltd., Nicosia, Cyprus a materials informatics company.

## Abbreviations

3D: Three-dimensional
AOP: Adverse outcome pathways
APD: Applicability domain
API: Application programming interface
CIF: Crystallographic information files
CV: Cross validation
GUI: Graphical user interface
FAIR: Findable, accessible, interoperable, re-useable
ICE: Individual conditional expectation
kNN: k-nearest neighbours
LOO: Leave-one-out
MAE: Mean absolute error
MHT: Magnetic hyperthermia
ML: Machine learning
MLP: Muli-layer perceptron
MRI: Magnetic resonance imaging
MSE: Mean squared error
NM: Nanomaterial
NP: Nanoparticle
PBPK: Physiologically based pharmacokinetic
PDP: Partial dependence plot
PEG: Polyethylene glycol
QMRF: QSAR model reporting format
QSAR: Quantitative structure–activity relationships
QSTR: Quantitative structure–toxicity relationship
RF: Random forest
RMSE: Root mean squared error
SbD: Safe by design
SGD: Stochastic gradient descent
SHAP: Shapley additive explanations
SR: Systematic review
XAI: Explainable artificial intelligence

## Supplementary Material

NA-008-D5NA00549C-s001

## Data Availability

Data for this article, including the unified dataset with the atomistic descriptors are available at the nanoPharos database at https://db.nanopharos.eu/Queries/Datasets.zul?datasetID=np29. Crystallographic Data for the three iron carbide phases were derived from the Crystallography Open Database (COD IDs: 1543664 (Fe_2_C), 1008725 (Fe_3_C), 1521831 (Fe_5_C_2_)).^97–99^ Supplementary information is available. See DOI: https://doi.org/10.1039/d5na00549c.
