# Supplementary material for: Atom-level descriptors and explainable prediction of iron carbide nanoparticles' cytotoxicity via the Enalos Cloud platform
Source: Nanoscale Adv. 2025 Nov 25;8(2):646–61. doi: 10.1039/d5na00549c (PMC12696484; doi:10.1039/d5na00549c)
Supplement: NA-008-D5NA00549C-s001 [file NA-008-D5NA00549C-s001.pdf]

Supplementary Information (SI) for Nanoscale Advances.
This journal is © The Royal Society of Chemistry 2025

Supporting Information

Atom-Level Descriptors and Explainable Prediction of Iron Carbide Nanoparticles' Cytotoxicity via the Enalos Cloud Platform

Maria Antoniou[1,2,3], Dimitra-Danai Varsou[3,4], Andreas Tsoumanis[1,3,4], Georgia Melagraki[5], Iseult Lynch[6], Antreas Afantitis[1,3,4*]

[1] Department of Nanoinformatics, NovaMechanics Ltd., Nicosia 1046, Cyprus;
[2] Computation-Based Science and Technology Research Center, The Cyprus Institute, Nicosia 2121, Cyprus;
[3] Entelos Institute, Larnaca 6059, Cyprus;
[4] Department of NanoInformatics, NovaMechanics MIKE, Piraeus, 18545, Greece;
[5] Division of Physical Sciences & Applications, Hellenic Military Academy, 16672 Vari, Greece;
[6] School of Geography, Earth and Environmental Sciences, University of Birmingham Edgbaston Birmingham 12 B15 2TT, United Kingdom;
* Corresponding author: afantitis@novamechanics.com

S1. Definition of Shapley Additive Explanations

For each feature *i* in a trained ML model *f*, the SHAP value φ_i is calculated by averaging the marginal contributions of all the permutations of the feature set. This can be expressed as:

$$\varphi_i(f,x) = \sum_{z' \subseteq x'} \frac{|z'|!(M - |z'| - 1)!}{M!}\left[f_x(z') - f_x(z'\backslash i)\right] \#(Eq.S1)$$

where:

- M is the total number of features,
- z' is a subset of features from the set x',
- x is an input datapoint to model *f*,
- $f_x(z')$ is the model output with the feature *i* present, and
- $f_x(z'\backslash i)$ is the model output with the feature *i* removed.

S2. Applicability Domain based on Gower distance

The Gower distance metric between observations *i* and *j* is defined as a weighted average of the distances on the different variables (*p*):

$$d_{ij} = \frac{\sum_{k=1}^{p} w_{ij,k} \cdot s_{ij,k}}{\sum_{k}^{p} s_{ij,k}} \#(Eq.S2)$$

where: w_{ij} is the weight between *i* and *j* for feature *k*, and s_{ijk} is the distance between *i* and *j* for feature *k*. s_{ijk} applies a different formula for numerical and categorical data types.

For numerical attributes, the distances s_{ij} are calculated as the absolute differences scaled to the range [0,1]:

$$s_{ij,k} = \frac{|x_{ik} - x_{jk}|}{\max(x_k) - \min(x_k)} \#(Eq.S3)$$

For categorical attributes, an equal/non-equal matching comparison is used as follows:

$$s_{ij,k} = \begin{cases} 0, & if\ x_{ik} = x_{jk} \\ 1, & if\ x_{ik} \neq x_{jk} \end{cases} \#(Eq.S4)$$

All pairwise Gower distances are ranging from 0 to 1, signifying identical signifying maximally dissimilar samples respectively. In this implementation, weights in Eq. S2 are assigned based on variable importance, with attributes 'Concentration' and 'Coating group' being assigned a

weight of two, and a weight of 1 assigned in the remaining descriptors. The gower python package (v.0.1.2) was used for distance calculations.

Table S1. Statistical measures used for performance evaluation.

Metric	Metric Formula
**External Validation:**	
Coefficient of determination (R^2)	$1 - \frac{\sum_{i=1}^{N}(y_i - \hat{y}_i)^2}{\sum_{i=1}^{N}(y_i - \underline{y})^2}$
Mean Absolute Error (MAE)	$\frac{1}{N}\sum_{i=1}^{N}\lvert y_i - \hat{y}_i\rvert$
Mean Square error (MSE)	$\frac{1}{N}\sum_{i=1}^{N}(y_i - \hat{y}_i)^2$
Root Mean Square error (RMSE)	$\sqrt{\frac{1}{N}\sum_{i=1}^{N}(y_i - \hat{y}_i)^2}$
Adjusted R^2	$1 - \frac{(1 - R^2)(N - 1)}{N - K - 1}$

Definitions for Golbraikh and Tropsha's criteria:

$$k = \frac{\sum_{i=1}^{N} y_i \hat{y}_i}{\sum_{i=1}^{N} \hat{y}_i^{\,2}}$$

$$k' = \frac{\sum_{i=1}^{N} y_i \hat{y}_i}{\sum_{i=1}^{N} y_i^{\,2}}$$

$$R_o^2 = 1 - \frac{\sum_{i=1}^{N}\left(\hat{y}_i - \hat{y}_i^{\,r_o}\right)^2}{\sum_{i=1}^{N}(\hat{y}_i - \underline{y})^2}, \ where\ \hat{y}_i^{\,r_o} = k'y$$

$$R_o^{'2} = 1 - \frac{\sum_{i=1}^{N}\left(y_i - y_i^{r_o}\right)^2}{\sum_{i=1}^{N}\left(y_i - \hat{\underline{y}}\right)^2}, where\ y_i^{r_o} = k'\hat{y}$$

N: the number of samples; y_i*: the actual endpoint values of the* i^{th} *sample;* $\hat{y}_i$ *the predicted endpoint values of the* i^{th} *sample;* $\underline{y}$*: the average true endpoint values;* $\hat{\underline{y}}$*: the average predicted endpoint values; K: the number of independent features in the model.*

Table S2: Five-fold cross validation performance using experimental evidence and atomistic-based descriptors. Results are reported as MAEcv ± standard deviation across folds.

Regression Algorithm	Experimental data (MAEcv ± std)	Atomistic descriptors (MAEcv ± std)
Random Forest Regressor (1)	0.073 ± 0.011	0.026 ± 0.008
k-Nearest Neighbours (2)	0.105 ± 0.012	0.039 ± 0.011
Linear SGD (3)	0.142 ± 0.007	0.043 ± 0.022
MLP Regressor (4)	0.161 ± 0.021	0.044 ± 0.019
LightGBM (5)	0.124 ± 0.015	0.038 ± 0.017
XGBoost Regressor (6)	0.067 ± 0.006	0.030 ± 0.009

Table S3: Hyperparameter optimisation for the six selected regression algorithms before and after using the calculated atomistic descriptors.

Regression Algorithm	Tested hyperparameter grids	Optimal Hyperparameters	
		Experimental data	Atomistic Descriptors
Random Forest Regressor (1)	Number of trees: [15, 25, 30, 40],<br>Max depth: [None, 2, 5, 10],<br>Min impurity decrease: [0.0, 0.05, 0.1]	N. trees: 40<br>Max depth: None<br>Min. imp decrease: 0.0	N. trees: 25<br>Max depth: 10<br>Min. imp decrease: 0.0
k-Nearest Neighbours (2)	Number of neighbours: [4, 5, 6, 7, 8],<br>Weight function: [uniform, distance]	N. neighbours: 4<br>Weights: distance	N. neighbours: 8<br>Weights: distance
Linear SGD (3)	Learning rate: [0.001, 0.05, 0.1],<br>Max iterations: [10, 20, 30, 40]	Learning rate: 0.05<br>Max iterations: 30	Learning rate: 0.05<br>Max iterations: 20
MLP Regressor (4)	Activation function: [logistic, rectified linear unit (ReLU)], Initial learning rate: [0.001, 0.005, 0.1, 0.5]	Act. function: logistic<br>Initial learning rate: 0.01	Act. function: logistic<br>Initial learning rate: 0.005
LightGBM (5)	Number of estimators: [15, 25, 40, 50]<br>Learning rate: [0.001, 0.05, 0.01]	N. estimators: 50<br>Learning rate: 0.05	N. estimators: 15<br>Learning rate: 0.05
XGBoost Regressor (6)	Learning rate: [0.2, 0.25, 0.3],<br>Max depth: [0.1, 0.01, 0.001]	Learning rate: 0.2<br>Max depth: None	Learning rate: 0.2<br>Max depth: 6

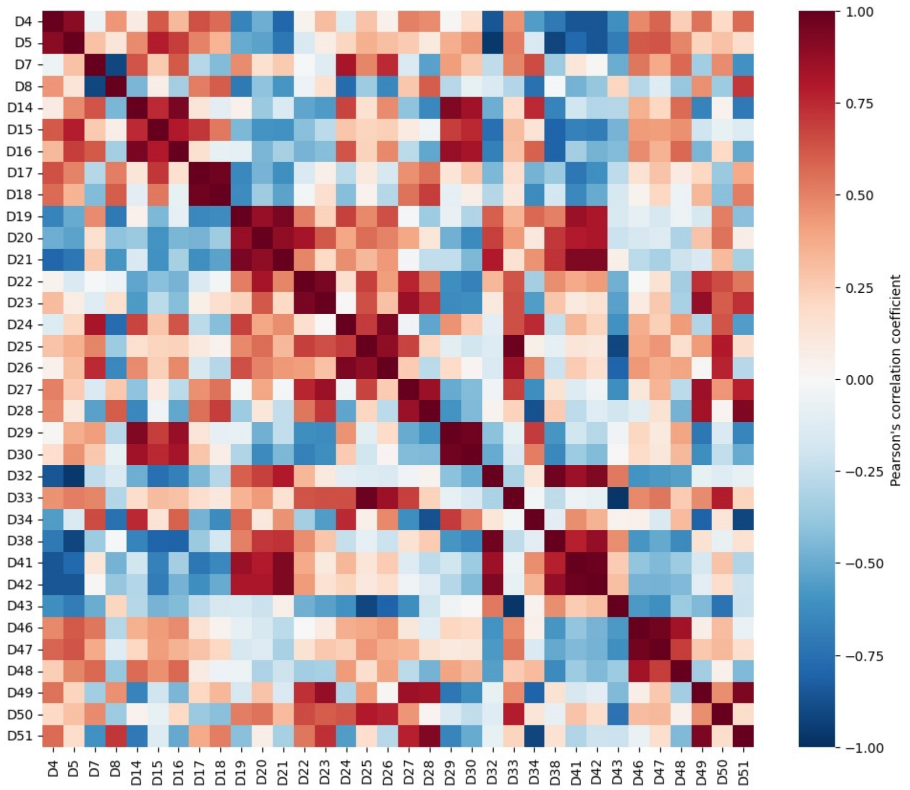

Figure S1: Pearson's correlation coefficient heatmap of the 33 atomistic descriptors remaining after the removal of highly correlated features (99%).

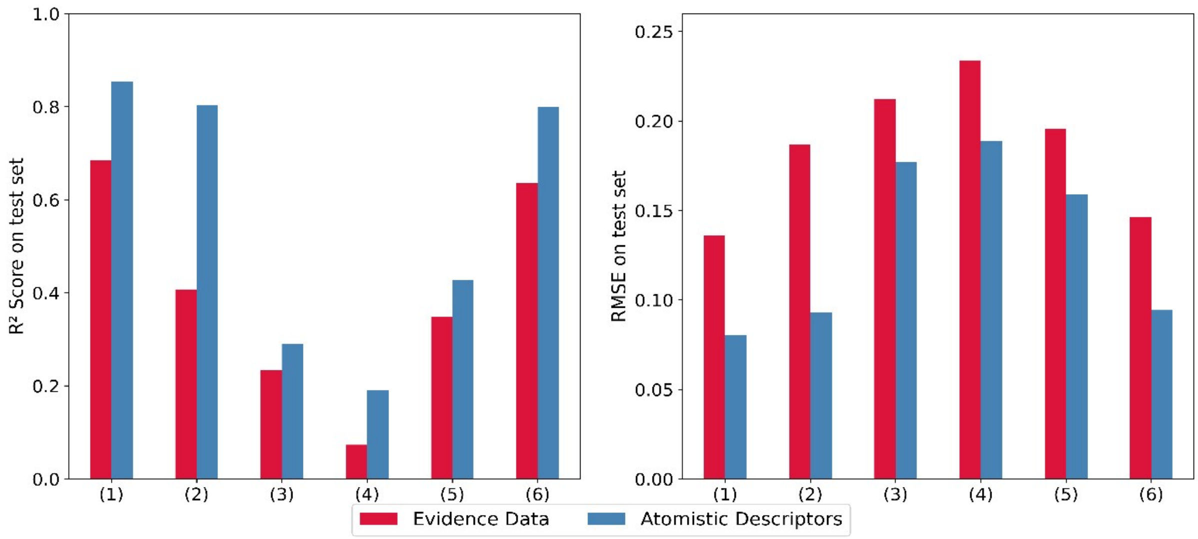

Figure S2: Performance comparison between the tuned regressor algorithms applied on the test set using the initial (red) and extended (blue) set of features. Each number in parentheses on the x-axis corresponds to the algorithms ordered as listed in Table S2.

S3. Dissemination of the QSTR for ICNP cytotoxicity using the QMRF reporting template

	Element	Explanation
**1.**	**QSAR identifier**	
1.1.	QSAR identifier (title)	NanoQSTR model for the quantitative prediction of iron carbide nanoparticles' (ICNP) *in vitro* cell viability.
1.2	Other related models	Not Applicable
1.3.	Software coding the model	Isalos Analytics Platform (v.0.4.0)<br>Model available via the Enalos CHIASMA Cloud Platform
**2.**	**General information**	
2.0	Abstract	A nanoinformatics model for the biosafety assessment of iron carbide nanoparticles (ICNPs, e.g., Fe_5C_2, Fe_3C), a promising material for biomedical applications. Model output is the viability of cells *in vitro* (as a %) following 24 hour exposure to ICNP. The model is based on physicochemical characteristics of the NPs and computationally derived properties on the atomistic level.
2.1.	Date of QMRF	-
2.2.	QMRF author(s) and contact details	Maria Antoniou (antoniou@novamechanics.com)<br>Andreas Tsoumanis (tsoumanis@novamechanics.com)<br>Georgia Melagraki (georgiamelagraki@gmail.com)<br>Iseult Lynch (i.lynch@bham.ac.uk)<br>Antreas Afantitis (afantitis@novamechanics.com)
2.3.	Date of QMRF update(s)	September 2025
2.4.	QMRF update(s)	Not Applicable
2.5.	Model developer(s) and contact details	Maria Antoniou – antoniou@novamechanics.com
2.6.	Date of model development and/or publication	Date of model publication: September 2025
2.7.	Reference(s) to main scientific papers and/or software package	Antoniou et al. 'Atom-Level Descriptors and Explainable Prediction of Iron Carbide Nanoparticles' Cytotoxicity via the Enalos Cloud Platform'<br>Software package: https://isalos.novamechanics.com/
2.8.	Availability of information about the model	The model is proprietary. The source code is not available; however, the model is implemented in a public web service. The model development process is fully described in the original publication, and the training and external validation datasets are provided as supplementary material and via the NanoPharos database:<br>https://db.nanopharos.eu/Queries/Datasets.zul?datasetID=np29
2.9.	Availability of another QMRF for exactly the same model	None

**3**	**Defining the endpoint - OECD Principle 1: “A DEFINED ENDPOINT"**	**PRINCIPLE 1: “A DEFINED ENDPOINT". ENDPOINT refers to any physicochemical, biological, or environmental property/activity/effect that can be measured and therefore modelled. The intent of PRINCIPLE 1 (a (Q)SAR should be associated with a defined endpoint) is to ensure clarity in the endpoint being predicted by a given model, since a given endpoint could be determined by different experimental protocols and under different experimental conditions. It is therefore important to identify the experimental system and test conditions that is being modelled by the (Q)SAR.**
3.1.	Species	Human and murine immortalised cell lines.<br>Normal cell lines included: HEK-293T, L929, NIH-3T3 and RAW264.7.<br>Cancer cell lines included: 4T1, A549, HeLa, MCF-7, MDA-MB-231, SK-BR-3, SK-OV-3 and U87MG.
3.2.	Endpoint	Cell viability as the cellular-level toxicity endpoint
3.3	Comment on endpoint	Cell viability represents the cell survival percentage following a 24-hour exposure to ICNPs. The target values were extracted from dose-dependent plots from relevant publications, with the process and results from the systematic review documented in Antoniou et al., 10.3390/nano14090734 [1]
3.4.	Endpoint units	Percentile values (numerical endpoint)
3.5.	Dependent variable	Not applicable
3.6.	Experimental protocol	Different cytotoxicity assays were used in the individual studies to assess the biosafety of ICNPs (MTT, SRB and CCK8 colorimetric indicator assays). In cellular metabolic activity assays, a decrease in the number of living cells results in a decrease in the metabolic activity of the tested sample. More information on the experimental protocols can be found in the respective research articles gathered in [1].
3.7.	Endpoint data quality and variability	A meta-analysis was also conducted that revealed high heterogeneity (I^2=77.1) in the dataset. A subgroup analysis was performed to study the factors that influence toxicity ($p<0.001$). The ToxRTool [2] was used to perform a quality evaluation of the experiments and assess the risk of bias. Detailed information on the meta-analysis and quality assessment on the studies can be found in [1].<br><br>Endpoint Variability: Mean: 82.8%, St. Dev: ±21.8%
**4**	**Defining the algorithm - OECD Principle 2 : “AN UNAMBIGUOUS ALGORITHM”**	**PRINCIPLE 2: “AN UNAMBIGUOUS ALGORITHM”. The (Q)SAR estimate of an endpoint is the result of applying an ALGORITHM to a set of structural parameters which describe the chemical structure. The intent of PRINCIPLE 2 (a (Q)SAR should be associated with an unambiguous algorithm) is to ensure transparency in the model algorithm that generates predictions of an endpoint from information on chemical structure and/or physicochemical properties. In this context, algorithm refers to any mathematical equation, decision rule or output approach.**
4.1.	Type of model	Ensemble learning algorithm: Random Forest

4.2.	Explicit algorithm	Random Forest is an ensemble learning method that operates by building multiple randomised decision trees during training and obtaining the prediction of the individual trees. The decision trees are constructed in parallel, with no interaction between them, using random subsets of training data and input attributes to ensure diversity. Predictions independently made by all the trees in the forest are aggregated and averaged in regression tasks.<br><br>Optimal hyperparameters after a nested Cross Validation scheme:<br>Number of trees = 25,<br>Maximum depth of trees = 10<br>Minimum impurity decrease = 0.0
4.3.	Descriptors in the model	Experiment-related Descriptors:<br>● Shell material<br>● Coating group<br>● Core NP size (nm) and Overall NP size (nm)<br>● Concentration (μg/L)<br>Atomistic Descriptors:<br>● The average potential energy of the core atoms (eV)<br>● The average difference of the potential energy between core and shell atoms (eV)<br>● The average coordination parameter (3Å of the core atoms<br>● The average coordination parameter (4Å) of the shell atoms<br>● Lattice energy of NP (eV)<br>● Lattice energy of bulk material - Lattice energy of NP (eV)<br>● The average second hex parameter of all atoms<br>● The average second hex parameter of the shell atoms
4.4.	Descriptor selection	From the initial dataset obtained after data mining, features that only described the biological evaluation (e.g., cell type, health status of the cell) were eliminated.<br>Zero-variance features were excluded and one feature from each highly correlated pair of attributes (i.e., Pearson correlation coefficient above 99%) were removed from the pool of 57 atomistic descriptors. Shapley additive explanations (SHAP) analysis was used to determine feature importance: a random forest (number of trees = 40, max depth=10) was trained on the dataset and a tree explained was built to obtain the mean absolute SHAP values for all features. The eight features with the highest SHAP values were selected as the most contributing atomistic descriptors.
4.5.	Algorithm and descriptor generation	Computational descriptors were generated from atomistic simulations of the nanoparticle cores using the size, shape and material phase (Fe_5C_2, Fe_3C and Fe_2C). The particles from the dataset were considered spherical in shape, as obtained from the TEM images of the individual studies.
4.6.	Software name and version for descriptor generation	Nanoparticle Construction (NanoConstruct) Tool Powered by Enalos RiskGONE Cloud Platform [3], http://enaloscloud.novamechanics.com/riskgone/nanoconstruct/<br>The crystallographic information files (CIF) of iron carbide phases were as follows:<br>Fe_5C_2: 1521831.cif, Fe_3C: 1008725.cif, Fe_2C: 1543664.cif.<br><br>Force Field key in OpenKIM:

		MEAM_LAMMPS_LiyanageKimHouze_2014_FeC__MO_075279800195_002
4.7.	Chemicals/Descriptors ratio	130 training NP samples / 13 descriptors
**5**	**Defining the applicability domain - OECD Principle 3: "A DEFINED DOMAIN OF APPLICABILITY"**	**PRINCIPLE 3: "A DEFINED DOMAIN OF APPLICABILITY". APPLICABILITY DOMAIN refers to the response and chemical structure space in which the model makes predictions with a given reliability. Ideally the applicability domain should express the structural, physicochemical and response space of the model. The CHEMICAL STRUCTURE (x variable) space can be expressed by information on physicochemical properties and/or structural fragments. The RESPONSE (y variable) can be any physicochemical, biological or environmental effect that is being predicted. According to PRINCIPLE 3 a (Q)SAR should be associated with a defined domain of applicability. Section 5 can be repeated (e.g., 5.a, 5.b, 5.c, etc) as many times as necessary if more than one method has been used to assess the applicability domain.**
5.1.	Description of the applicability domain of the model	A distance-based method is used for the domain of applicability definition. An applicability domain threshold (APD) is defined by fixed boundaries, and then the distance of a new sample is compared to APD to assess the reliability of the new prediction.
5.2.	Method used to assess the applicability domain	The predefined applicability domain threshold is calculated as:<br>$APD = \langle d \rangle + Z\sigma$<br>where d and σ are the average and the standard deviation of the distances respectively, and z is an empirical parameter to control the significance level, whose default value is 0.5. In case that the distance from an external compound to its nearest neighbour is larger than the APD threshold, then the prediction falls outside of the applicability domain, and it is labelled as unreliable.<br>Both Euclidean and Gower distances are used as distance metrics to assess proximity.<br>When Euclidean distances are considered, categorical features (i.e. shell and coating material) are not included in the APD limit calculation.<br>When Gower distances are considered, weights of 2 are assigned to features 'concentration' and 'coating groups', while the remaining features are assigned a weight of 1.
5.3.	Software name and version for applicability domain assessment	- Isalos Analytics Platform (v.0.4.0) "Domain-APD" function for Euclidean distance<br>- Python package gower (v.0.1.2) for Gower distance
5.4.	Limits of applicability	$APD,_{Euclidean}$ = 96.307<br>$APD,_{Gower}$ = 0.2652
**6**	**Defining goodness-of-fit and robustness (internal validation) – OECD Principle 4: "APPROPRIATE MEASURES OF GOODNESS-OF-FIT, ROBUSTENESS AND**	**PRINCIPLE 4: "APPROPRIATE MEASURES OF GOODNESS-OF-FIT, ROBUSTENESS AND PREDICTIVITY". PRINCIPLE 4 expresses the need to perform validation to establish the performance of the model. GOODNESS-OF-FIT and ROBUSTNESS refer to the internal model performance.**

	**PREDICTIVITY”**	
6.1.	Availability of the training set	The training set is available at the NanoPharos database at the link: https://db.nanopharos.eu/Queries/Datasets.zul?datasetID=np29 and via an application programming interface: https://db.nanopharos.eu/swagger-ui/ (datasetID=np29)
6.2.	Available information for the training set	a) ICNP formulations including their physicochemical characterisation<br>b) Pool of 57 atomistic descriptors<br>c) Data on *in vitro* toxicity to immortalised cell lines
6.3.	Data for each descriptor variable for the training set	The training set including the atomistic descriptor values are available at NanoPharos. Two features with categorical attributes from the training set were converted to numerical values using ordinal encoding:<br>● Shell material (Carbon, Fe_3O_4, MnO_2, SiO_2, None)<br>● Coating group (None, PEG-based, protein-based, other)
6.4.	Data for the dependent variable for the training set	The dependent variable values of the training set are available at the extended dataset at the NanoPharos database (6.1).
6.5.	Other information about the training set	The k=186 samples of the entire dataset were randomly split into 70:30 proportions to form the training and test sets, corresponding to 130 and 56 indices respectively. A decrease in cell viability to less than 70% of the control sample is indicative of cytotoxic potential according to the ISO-10993-5(2009) definition for the performance of colorimetric activity assays. Since 18.3% of the samples in the entire dataset are considered ‘Toxic’, a similar proportion of instances exhibiting cell viability lower than 70% was kept in the training dataset (k=24 samples).
6.6.	Pre-processing of data before modelling	Two features with categorical attributes from the training set were converted to numerical values using ordinal encoding:<br>● Shell material: 0-Carbon, 1-Fe_3O_4, 2-MnO_2, 3-None, 4-SiO_2<br>● Coating group: 0-None, 1-Other, 2-PEG-based, 3-protein-based
6.7.	Statistics for goodness-of-fit	Statistics for goodness-of-fit:<br>● R^2 = 0.888<br>● Adjusted R^2 = 0.859<br>● Mean Absolute Error =0.044<br>● Mean Squared Error (MSE) = 0.005<br>● Root Mean Squared Error = 0.074
6.8.	Robustness - Statistics obtained by leave-one-out cross-validation	Statistics obtained by leave-one-out cross validation on the training set:<br>LOOcv R^2: 0.639<br>LOO_{CV} MAE: 0.091<br>LOO_{CV} MSE: 0.0189<br>LOO_{CV} RMSE: 0.09

6.9.	Robustness - Statistics obtained by leave-many-out cross-validation	A nested 5-fold cross validation scheme was applied for hyperparameter tuning (random selection):<br>MAE_{5cv} = 0.026 ± 0.008
6.10.	Robustness - Statistics obtained by Y-scrambling	Statistics obtained for 5 randomisations with y-scrambled endpoint values:<br>Rand1: R^2=0.0198, MAE=0.1521, RMSE=0.2080<br>Rand2: R^2=0.0610, MAE=0.1426, RMSE=0.2036<br>Rand3: R^2=0.0221, MAE=0.1530, RMSE=0.2078<br>Rand4: R^2=0.0728, MAE=0.1497, RMSE=0.2023<br>Rand5: R^2=0.0381, MAE=0.1547, RMSE=0.2061
6.11.	Robustness - Statistics obtained by bootstrap	Not Applicable
6.12.	Robustness - Statistics obtained by other methods	Golbraikh and Tropsha's criteria for predictive modelling:<br>● R^2 = 0.844 > 0.6<br>● $Qloo^2$ = 0.639 > 0.5<br>● r^2-Ro^2/r^2 = 0.082 < 0.1<br>● r^2-Ro'^2/r^2 = -0.001 < 0.1<br>● $\lvert Ro^2$-$Ro'^2\rvert$ = 0.068 < 0.3<br>● 0.85 < k=0.994 < 1.15<br>● 0.85 < k'=0.995 < 1.15
**7**	**Defining predictivity (external validation) – OECD Principle 4: "APPROPRIATE MEASURES OF GOODNESS-OF-FIT, ROBUSTENESS AND PREDICTIVITY"**	**PRINCIPLE 4: "APPROPRIATE MEASURES OF GOODNESS-OF-FIT, ROBUSTENESS AND PREDICTIVITY". PRINCIPLE 4 expresses the need to perform validation to establish the performance of the model. PREDICTIVITY refers to the external model validation. Section 7 can be repeated (e.g., 7.a, 7.b, 7.c, etc) as many times as necessary if more validation studies need to be reported in the QMRF.**
7.1.	Availability of the external validation set	The test set is available at the NanoPharos database at the link: https://db.nanopharos.eu/Queries/Datasets.zul?datasetID=np29 and via an application programming interface: https://db.nanopharos.eu/swagger-ui/ (datasetID=np29)
7.2.	Available information for the external validation set	a) ICNP formulations including their physicochemical characterisation<br>b) Pool of 57 atomistic descriptors<br>c) Data on *in vitro* toxicity to immortalised cell lines
7.3.	Data for each descriptor variable for the external validation set	The test set including the atomistic descriptor values are available at NanoPharos.
7.4.	Data for the dependent variable for the external validation set	The dependent variable values of the validation set are available at the extended dataset at the NanoPharos database (see 7.1.).
7.5.	Other information about the external validation set	Not Applicable
7.6.	Experimental design of test set	30% of the dataset that was not involved in the training process was retained for model validation purposes.

7.7.	Predictivity - Statistics obtained by external validation	Statistics for goodness-of-fit:<br>● R^2 = 0.844<br>● Adjusted R^2 = 0.704<br>● Mean Absolute Error =0.063<br>● Mean Squared Error (MSE) = 0.007<br>● Root Mean Squared Error = 0.083
7.8.	Predictivity - Assessment of the external validation set	The test set is sufficient in size, since it represents a significant part (30%) of the initial dataset. The distribution of cell viability values was ensured by using stratified sampling to divide the dataset into two representative subsets. The two subsets exhibit similar variability of the target endpoint:<br>Training set: Mean=83.1%, StDev=22.1%<br>Test set: Mean=82.2%, StDev=21.0%<br>All the treatments from the hold-out set fell inside the applicability domain limits.
7.9.	Comments on the external validation of the model	Other metrics used: External explained variance coefficient<br>$Q_{ext}^2 = 1 - \frac{\sum_{i=1}^{N}(y_i - \hat{y}_i)^2}{\sum_{i=1}^{N}(y_i - \bar{y}_{train})^2} = 0.753$<br><br>External validation on a blind set from an independent study. Castellano-Soria et al. [5] assessed the biocompatibility of spherical Fe_3C@C core–shell ICNPs (Fe_3C phase: *Pnma62*, graphite coating ~2.5 nm) using a similar experimental protocol as the training data (24-hour exposure on MCF-7 tumour cells, treatment of NPs at a surfactant proportion of 5 mmol) at four concentrations between 12.5 and 100 µg/mL).<br>Validation metrics: R_{ext}^2 = 0.517, MAE_{ext}=0.033, MSE_{ext}=0.002, $RMSE_{ext}$=0.047. 95% confidence intervals were estimated via bootstrapping.

#	Actual	Pred.	Abs. error	95% Confidence Intervals
1	99.8%	90.6%	9.2%	[0.924, 0.990]
2	94.1%	92.4%	1.7%	[0.876, 1.009]
3	87.2%	88.4%	1.2%	[0.877, 1.003]
4	81.5%	82.6%	1.1%	[0.787, 0.905]

All samples in the blind set were considered reliable for both APD methods.

# sample	Domain (Euclidean)	Domain (Gower)
1	44.493 (+)	0.2186 (+)
2	44.423 (+)	0.2182 (+)
3	43.929 (+)	0.2182 (+)
4	43.880 (+)	0.2181 (+)

**8**	**Providing a mechanistic interpretation - OECD Principle 5: "A**	**PRINCIPLE 5: "A MECHANISTIC INTERPRETATION, IF POSSIBLE". According to PRINCIPLE 5, a (Q)SAR should be associated with a mechanistic interpretation, if possible.**

	**MECHANISTIC INTERPRETATION, IF POSSIBLE"**	
8.1.	Mechanistic basis of the model	Before modelling, a subgroup analysis was performed to find statistical correlations between the toxicity of ICNPs and factors such as the particle size, cell species, and cell health status. The type of conjugated ligand on the ICNP and NP concentration range are statistically significant factors influencing cytotoxicity ($p<0.001$).<br><br>Experimental-related descriptors: Shell material and coating groups determine the ICNP's surface chemistry and influence their nano-bio interactions, affecting toxicity. NP size affects their surface-to-volume ratios and determines NP permeability through cell membranes. Lastly, higher NP concentration ranges have an adverse response to biological systems.<br><br>Atomistic descriptors: The average potential energy of core atoms and potential energy difference between core and shell atoms were selected due to their correlation to the ICNP structure's stability. Lower potential energies are indicative of more stable structures, while large differences between the two layers of atoms reflect potential instabilities. Descriptors calculated as the differences between core and surface atom properties are relevant to surface reactivity. The lattice energy of a NP and its difference from that of the bulk materials are descriptive of the particles' stability as well. In addition, coordination parameters (3Å for core and 4Å for shell) provide insights into ICNP's atomic arrangements by calculating the average number of neighbouring atoms of a single atom [4].<br><br>SHAP analysis (python library, v.0.45.1) was used as a post-analysis method to explain model output in understandable terms and describe how each NP property influences predictions.
8.2.	A priori or a posteriori mechanistic interpretation	A priori (meta-analysis), and<br>A posteriori mechanistic interpretation (SHAP analysis, PDP and ICE plots, permutation feature importance)
8.3.	Other information about the mechanistic interpretation	Beeswarm plots are generated to visualise findings for each tested treatment.
**9**	**Miscellaneous information**	
9.1.	Comments	Not applicable
9.2.	Bibliography	[1] Antoniou, M.; Melagraki, G.; Lynch, I.; Afantitis, A. In Vitro Toxicological Insights from the Biomedical Applications of Iron Carbide Nanoparticles in Tumor Theranostics: A Systematic Review and Meta-Analysis. *Nanomaterials* 2024, *14* (9), 734<br><br>[2] Schneider, K.; Schwarz, M.; Burkholder, I.; Kopp-Schneider, A.; Edler, L.; Kinsner-Ovaskainen, A.; Hartung, T.; Hoffmann, S. "ToxRTool", a New Tool to Assess the Reliability of Toxicological Data. *Toxicology Letters* 2009, *189* (2), 138–144.<br><br>[3] Kolokathis, P. D.; Zouraris, D.; Voyiatzis, E.; Sidiropoulos, N. K.; Tsoumanis, A.; Melagraki, G.; Tämm, K.; Lynch, I.; Afantitis, A. NanoConstruct: A Web Application Builder of Ellipsoidal

		Nanoparticles for the Investigation of Their Crystal Growth, Stability, and the Calculation of Atomistic Descriptors. *Computational and Structural Biotechnology Journal* 2024, *25*, 81–90.<br><br>[4] Kirchhoff, B.; Jung, C.; Gaissmaier, D.; Braunwarth, L.; Fantauzzi, D.; Jacob, T. *In Silico* Characterization of Nanoparticles. *Phys. Chem. Chem. Phys.* 2023, *25* (19), 13228–13243<br><br>[5] A. Castellano-Soria, R. López-Méndez, A. Espinosa, C. Granados-Miralles, M. Varela, P. Marín, E. Navarro and J. López-Sánchez, Mater Today Chem, 2024, 39, 102143.
9.3	Supporting information	Not Applicable
